# ASPIRE‐FTD Study Update: A Phase 1/2 Clinical Study to Evaluate AVB‐101 in FTD with GRN Mutations

**DOI:** 10.1002/alz70859_102193

**Published:** 2025-12-25

**Authors:** Yi‐Ge Huang, Jane YC Chan, Rachel Sartaj, Christopher M Powell, Miroslaw Zabek, Gabriela Klodowska, Marcin Ratajczak, David L Cooper

**Affiliations:** ^1^ AviadoBio Ltd., London United Kingdom; ^2^ Mazowiecki Szpital Bródnowski, Warsaw Poland; ^3^ Neuro‐Care Centrum Medyczne, Katowice Poland; ^4^ EuroMedis, Szczecin Poland

## Abstract

**Background:**

The ASPIRE‐FTD study aims to evaluate the safety, tolerability and preliminary efficacy of AVB‐101 in patients with frontotemporal dementia due to progranulin mutations (FTD‐GRN). AVB‐101 uses an adeno‐associated virus 9 vector to deliver a functional copy of the GRN gene to thalamic neurons, which secrete and distribute progranulin (PGRN) throughout the brain. Direct intrathalamic delivery of AAV gene therapy has potential to achieve broad cortical biodistribution of PGRN, overcoming the challenges associated with the blood‐brain‐barrier and pial membrane, while using relatively low AAV doses versus intrathecal delivery. Here, we report preliminary safety from the first completed cohort.

**Method:**

This ongoing Phase 1/2 open‐label, ascending dose study (NCT06064890) will evaluate up to three dose levels of AVB‐101 in the dose escalation part, and further subjects may be dosed in the expansion part to provide additional safety and efficacy data. All subjects will receive a one‐time, bilateral intrathalamic infusion of AVB‐101 under real‐time magnetic resonance imaging (MRI) guidance. The primary objective is to evaluate safety and tolerability of AVB‐101: safety measures include adverse events (AEs) (related or not to AVB‐101 and the administration procedure), change from baseline in MRI results and clinical/laboratory assessments. Neurofilament light (NfL) protein in plasma and cerebrospinal fluid (CSF) is also measured.

**Results:**

AVB‐101 was well‐tolerated in all three patients at the dose level tested in this initial cohort. No clinically significant safety findings have been observed through the follow‐up period of up to 39, 26 and 12 weeks respectively for the three dosed subjects to date. Serial safety MRI demonstrated no significant hemorrhage, edema or inflammation.

There have been no serious AEs reported to date and no AEs related to AVB‐101 or the neurosurgical procedure. Additionally, neither prophylactic nor reactive immunosuppression has been required for any subject.

As expected, an early transient peak in serum and CSF NfL protein expression was observed after AVB‐101 administration, with trend to baseline thereafter.

**Conclusion:**

Preliminary data from the ongoing ASPIRE‐FTD trial suggests a favorable safety profile for AVB‐101 and the administration procedure. Recruitment is ongoing for the subsequent escalated dose cohort.

